# TRPing on Cell Swelling - TRPV4 Senses It

**DOI:** 10.3389/fimmu.2021.730982

**Published:** 2021-09-20

**Authors:** Trine L. Toft-Bertelsen, Nanna MacAulay

**Affiliations:** Department of Neuroscience, University of Copenhagen, Copenhagen, Denmark

**Keywords:** TRPV4 (transient receptor potential vanilloid 4), volume-sensitive channels, volume regulation, osmo-sensing, aquaporins (AQPs)

## Abstract

The transient receptor potential vanilloid 4 channel (TRPV4) is a non-selective cation channel that is widely expressed and activated by a range of stimuli. Amongst these stimuli, changes in cell volume feature as a prominent regulator of TRPV4 activity with cell swelling leading to channel activation. In experimental settings based on abrupt introduction of large osmotic gradients, TRPV4 activation requires co-expression of an aquaporin (AQP) to facilitate such cell swelling. However, TRPV4 readily responds to cell volume increase irrespectively of the molecular mechanism underlying the cell swelling and can, as such, be considered a sensor of increased cell volume. In this review, we will discuss the proposed events underlying the molecular coupling from cell swelling to channel activation and present the evidence of direct *versus* indirect swelling-activation of TRPV4. With this summary of the current knowledge of TRPV4 and its ability to sense cell volume changes, we hope to stimulate further experimental efforts in this area of research to clarify TRPV4’s role in physiology and pathophysiology.

## Cellular Detection of Volume Changes

Accurate and rapid sensing of the surrounding environment is key to survival for cells and organisms. Upon exposure to challenges that alter the cell volume, cellular regulatory mechanisms required for an appropriate physiological reaction to the condition causing cell swelling or shrinkage are set in motion.

### The Discovery of TRPV4

Detection of cell volume changes is perceived through sensory mechanisms, one of which was characterized in the invertebrates *Drosophila melanogaster* and *Caenorhabditis elegans* (1). Organisms with mutations in *osm-9* or *ocr-2*, which are genes encoding ion channels belonging to the transient receptor potential (TRP) channel superfamily, the vanilloid subfamily (TRPV) (2–4), were unable to produce cellular responses to stimuli leading to cell volume changes (1, 5). Such genes had at that point not been identified in vertebrate cells, and the search for mammalian homologues of *osm-9* was on.

In the year 2000, an ion channel was described that related to *osm-9* and VRL-1 (vanilloid receptor-like 1 protein, or TRPV2, a member of the vanilloid subfamily) and was gated by osmotic challenges (6). This ion channel, now known as TRPV4, was, using a combination of *in silico* analysis of expressed sequence tag databases and conventional molecular cloning, isolated as a novel vanilloid-like receptor from the human kidney (7). At the time, the channel was named VRL-2 due to its resemblance to VRL-1 (or TRPV2), a homologue of the capsaicin receptor, a heat-activated ion channel in the pain pathway (8) with a high threshold for noxious heat, and later known as VR-OAC (*v*anilloid *r*eceptor *r*elated *o*smotically *a*ctivated *c*hannel) (6). VRL-2 was subsequently identified in mouse, chicken and rat (6, 7, 9, 10).

#### The TRP Family and Biophysical Properties

The TRP superfamily is grouped into six major subfamilies based on nucleotide sequence homology: TRPA (ankyrin); TRPC (canonical); TRPM (melastin); TRPML (mucolipin); TRPP (polycystin) and TRPV (vanilloid), the latter of which can further be subdivided into six isoforms (TRPV1-6). TRPV4 has 871 amino acid residues and topological features of the channel are six transmembrane spanning segments (S1-S6), a re-entrant pore forming loop between S5-S6, intracellular N- and C-termini, and ankyrin domains in the cytosolic N-terminus (11). The channel preferentially forms homomers (12), although heteromers may occur with other members of the TRP superfamily (13–15). Biophysically, TRPV4 is characterized as a non-selective cation channel with a moderately high Ca^2+^ permeability ratio of PCa/PNa = 6-10 (16–18) with two aspartate residues (Asp^672^ and Asp^682^) dictating the Ca^2+^ selectivity of the TRPV4 pore (16). Cryo-EM studies demonstrated that the narrowest part of the TRPV4 selectivity filter had a wider diameter than the pore of the open TRPV1 channel (19). In addition, TRPV4 appears to lack an extracellular gate (19), which, taken together, allows for a broader variety of permeant ions (20). It remains unresolved whether the reported physiological TRPV4 activators work through the selectivity filter of TRPV4 to activate the channel (20).

#### TRPV4 as an Osmo-Sensor

TRPV4 was defined as a nonspecific cation channel gated by osmotic stimuli (2–4) and characterized as such as such from a study done in TRPV4-transfected CHO cells (21). The cells were exposed to osmotic challenges of ± 110 mOsm, and a robust Ca^2+^ transient was observed within seconds of a cell volume increase. Such hyposmotically-induced gating was proposed to take place *via* subtle changes in membrane tension (22, 23). Swelling-induced activation of TRPV4-mediated Ca^2+^ influx was shortly thereafter confirmed in HEK293 cells expressing ‘OTRPC4’ (*osm9*-like *t*ransient *r*eceptor *p*otential *c*hannel, member *4*, another name for TRPV4) (9). Hence, TRPV4 was set forward as an osmo-sensor activated by hyposmolar stress. The physiological impact of TRPV4-mediated osmosensing was demonstrated by the impaired regulation of systemic tonicity in mice genetically devoid of TRPV4 (24, 25). The dysregulation of the systemic fluid homeostasis in the TRPV4^-/-^ mice arose, at least in part, from impaired osmosensing in the circumventricular organ of the lamina terminalis and associated modification of antidiuretic hormone (ADH) secretion into the blood (24, 25). The TRPV4^-/-^ mice thus displayed lesser water intake (24, 25) and, in addition, presented with enlarged bladder capacity as a consequence of impaired stretch and pressure sensing in the bladder wall (25, 26). TRPV4 has, in addition, been implicated in pulmonary edema formation, partly *via* the observed down regulation of the co-localized AQP5 in the pulmonary epithelium obtained from TRPV4^-^/^-^ mice (27). Tissue obtained from meningioma patients demonstrated AQP4/TRPV4 co-expression in both edematous and non-edematous meningiomas, although in the surrounding peri-meningioma tissue, only AQP4 was upregulated (28). TRPV4 thus appears to be involved in physiological and pathophysiological processes involving fluid dynamics, in addition to its roles in skeletal dysplasias [for review of TRPV4 in pathology, see (29)]. However, the coupling between cell volume regulation and TRPV4 activity remains elusive.

#### TRPV4 Is a Genuine Sensor of Cell Volume Dynamics

**Figure 1:**
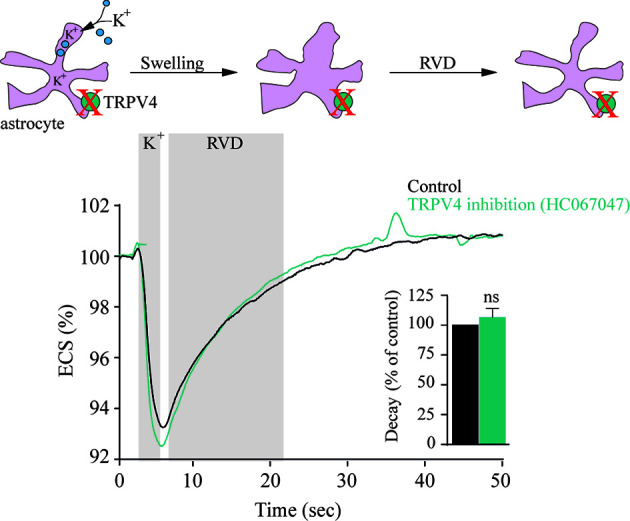
TRPV4 does not modulate astrocytic regulatory volume decrease following activity evoked astrocyte volume dynamics. Electrical stimulation of acute hippocampal slices from rats results in neuronal activity associated with a [K^+^]_o_ transient that leads to a brief change in cell volume of nearby astrocytic structures without application of an osmotic gradient to the test solution. Graphs illustrate a representative recording and summarized volume decay rates of the activity-evoked extracellular space dynamics in the absence or presence of a TRPV4 inhibitor [1 µM HC067047, same results obtained with the less specific TRPV4 inhibitor ruthenium red (1 µM)]. ns, not significant. Modified from (37) with permission.

Since the initial findings, swelling-induced activation of TRPV4 has been further documented upon heterologous expression of TRPV4 in yeast (30, 31) and in *Xenopus laevis* oocytes (30, 32, 33). In its native setting in retinal cells, TRPV4 responded to cell swelling with slow-onset, but sustained, activity in Müller glia, whereas in retinal ganglion neurons, TRPV4 responded with fast, but brief, bursts of activity (33, 34). Astrocytes respond to hyposmotically-induced cell swelling with TRPV4-mediated Ca^2+^ dynamics, which were proposed to be implicated in the subsequent regulatory volume decrease (35). However, during a more physiologically relevant astrocytic volume transient, as that observed during neuronal activity (in the absence of an experimentally-inflicted osmotic challenge) (36), the regulatory volume decrease was unaffected by TRPV4 inhibition, **Figure 1** (37). The molecular coupling between the altered osmolarity of the extracellular fluid and activation of TRPV4 was proposed to require the presence of an aquaporin, possibly even of a certain isoform: In renal cells; AQP2 (38), in salivary glands; AQP5 (39), and in astrocytes; AQP4 (35, 40, 41). However, these conclusions arose from experimental approaches based on abrupt exposure of the TRPV4-expressing cells to excessively large osmotic gradients of 100-250 mOsm. Such osmotic gradients will rarely, if ever, be observed outside the kidney in physiology or even pathophysiology – and not as an abruptly arising challenge. Still, the introduction of such non-physiological osmotic challenges is a common manner of experimental induction of cell volume changes for reasons of technical ease. Under such experimental conditions, *the rate* with which the cells swell upon an introduced osmotic challenge will depend on expression of an AQP of any isoform. Experiments employing such osmotic gradients will thus favor a concept of TRPV4 requiring the presence of an AQP to respond to a volume change (21, 32, 35, 39), see (37) for discussion of technical challenges with such experimental approaches. Notably, with smaller osmotic challenges (of the order of 20-40 mOsm) that promote cell swelling of a more physiological caliber, TRPV4-mediated Ca^2+^ dynamics vanished from retinal ganglion cells, but persisted in the Muller glia (33).

**Figure 2:**
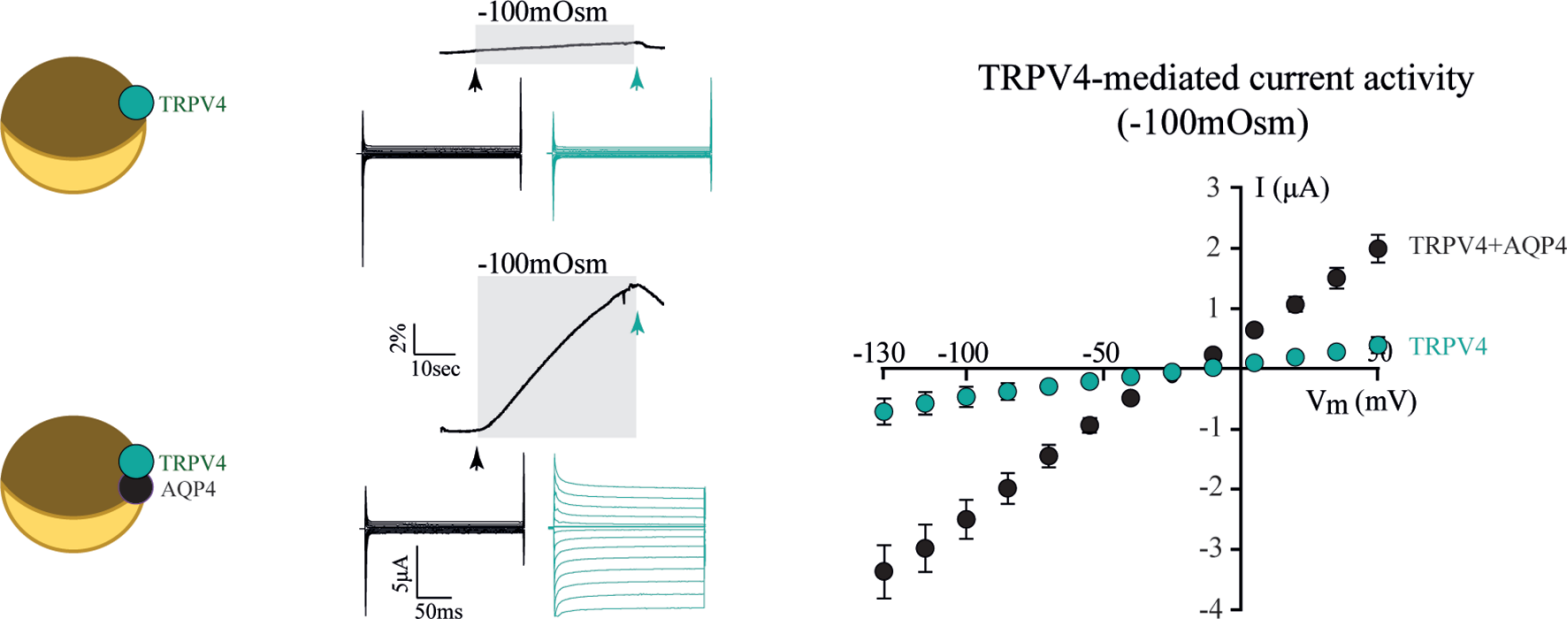
TRPV4 is activated by increased cell volume. Oocytes expressing TRPV4 alone (top traces) did not swell when exposed to a hyposmotic gradient (-100 mOsm) and did not respond with TRPV4-mediated currents during this challenge. Oocytes co-expressing TRPV4 and AQP4 (bottom traces) responded to the osmotic challenge with an abrupt volume increase and a resultant large membrane current (summarized in right panel). Modified from (32) with permission.

**Figure 3:**
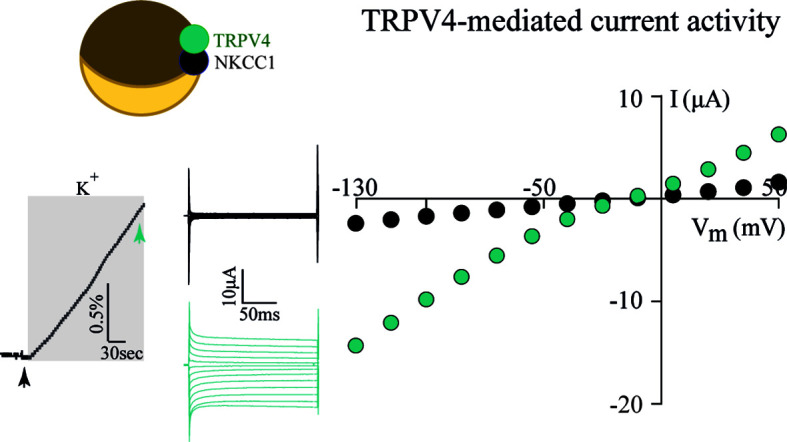
TRPV4 is activated by cell swelling, independently of AQPs and osmotic gradients. The water-transporting cotransporter NKCC1, co-expressed with TRPV4 in *Xenopus* oocytes, was activated by exposure to K^+^ (15 mM, equimolar replacement of Na^+^). This transporter activation led to a rapid volume increase (left panel) in the absence of an external introduction of an osmotic gradient. This cell volume increase promoted TRPV4 activation in the form of TRPV4-mediated currents (middle and right panels). Modified from (32) with permission.

To resolve the ability of TRPV4 to sense altered osmolarity *versus* simply the resulting cell changes, TRPV4 was heterologously expressed in *Xenopus laevis* oocytes with notoriously low intrinsic water permeability, either alone or co-expressed with an AQP (32). Introduction of a hyposmotic challenge led to abrupt cell swelling in the TRPV4-AQP-expressing oocytes and a resulting TRPV4-mediated membrane current, irrespective of the AQP isoform (32). None of these observations were detected in oocytes expressing TRPV4 alone (in the absence of an AQP), demonstrating that TRPV4 responded to the cell volume increase rather than the introduced osmotic challenge itself, **Figure 2** (32). These data are consistent with other reports in cortical and retinal glia, concluding that membrane expression of an AQP permitted a rapid cell swelling upon experimentally-inflicted osmotic challenges and thus allowed TRPV4 to respond to the resulting abrupt cell swelling (41, 42). This notion was cemented by a demonstration that swelling of TRPV4-expressing oocytes achieved without introduction of an osmotic challenge and in the absence of AQP co-expression sufficed to activate TRPV4, **Figure 3** (32). Such oocyte cell swelling was achieved by co-expression of a water-translocating cotransporter, the Na^+^, K^+^, 2Cl^-^ cotransporter (NKCC1), which upon activation leads to cell swelling by inward transport of its substrates along with a fixed number of water molecules (43, 44). TRPV4 is thereby established as a genuine volume‐sensor, rather than an osmo‐sensor (32), possibly induced by the membrane stretch achieved as a consequence of cell swelling (6, 24, 45). At the time, the molecular mechanisms coupling cell swelling to TRPV4 channel opening remained obscure.

## From Cell Swelling to TRPV4 Activation

TRPV4 represents a sensor of cell swelling. The underlying molecular link between cell swelling and channel opening has proven elusive, but can occur either directly or *via* an indirect pathway of cellular modulators.

### Direct Coupling of Cell Volume Changes to TRPV4 Activation

#### TRPV4 Gating *via* Mechanical Probing *Versus* Cell Volume Increase

Cell swelling may modulate TRPV4 gating in a more or less direct manner, or the resulting membrane stretch may serve as a mechanical disturbance that could be distinguished from the cellular volume dynamics. Various experimental strategies have been employed to distinguish the two, i.e. stretching of the cell membrane in the absence of a volume change (46–48) which has been employed to demonstrate (49–51) or not to demonstrate (9, 52, 53) direct activation of TRPV4 by mechanical probing. It therefore remains unresolved to what extent TRPV4 activation occurs by direct mechanical probing, rather than as a consequence of the cell volume changes.

#### TRPV4 Gating *via* Coupling to Cytoskeletal Components

A direct coupling of cell swelling to channel activation could be obtained by a tethering of intracellular components of TRPV4 to the cytoskeleton. Such coupling could provide the swelling-induced mechanical impact on the channel required to promote channel opening. TRPV4 has been demonstrated to co-localize with cytoskeletal components such as actin, microtubules, and microfilaments (54–56), with a specific binding site for F-actin in the TRPV4 N-terminus (55). Modulation of actin, *via* manipulation of the β1-integrins that couple the extracellular matrix and actin filaments, promoted TRPV4 activity (57). Inhibition of cytoskeletal rearrangements disrupted actin-TRPV4 co-localization (58) and reduced TRPV4 activity (54, 55) in a manner that did not affect cell swelling-induced TRPV4-activation (33). Cytoskeletal tethering of TRPV4 thus affects TRPV4 activity and therefore most likely also its volume regulation, although dynamic rearrangements within the cytoskeleton are not required for the swelling-induced channel activation (33).

#### TRPV4 Gating *via* Its N-Terminal Volume Sensor

**Figure 4:**
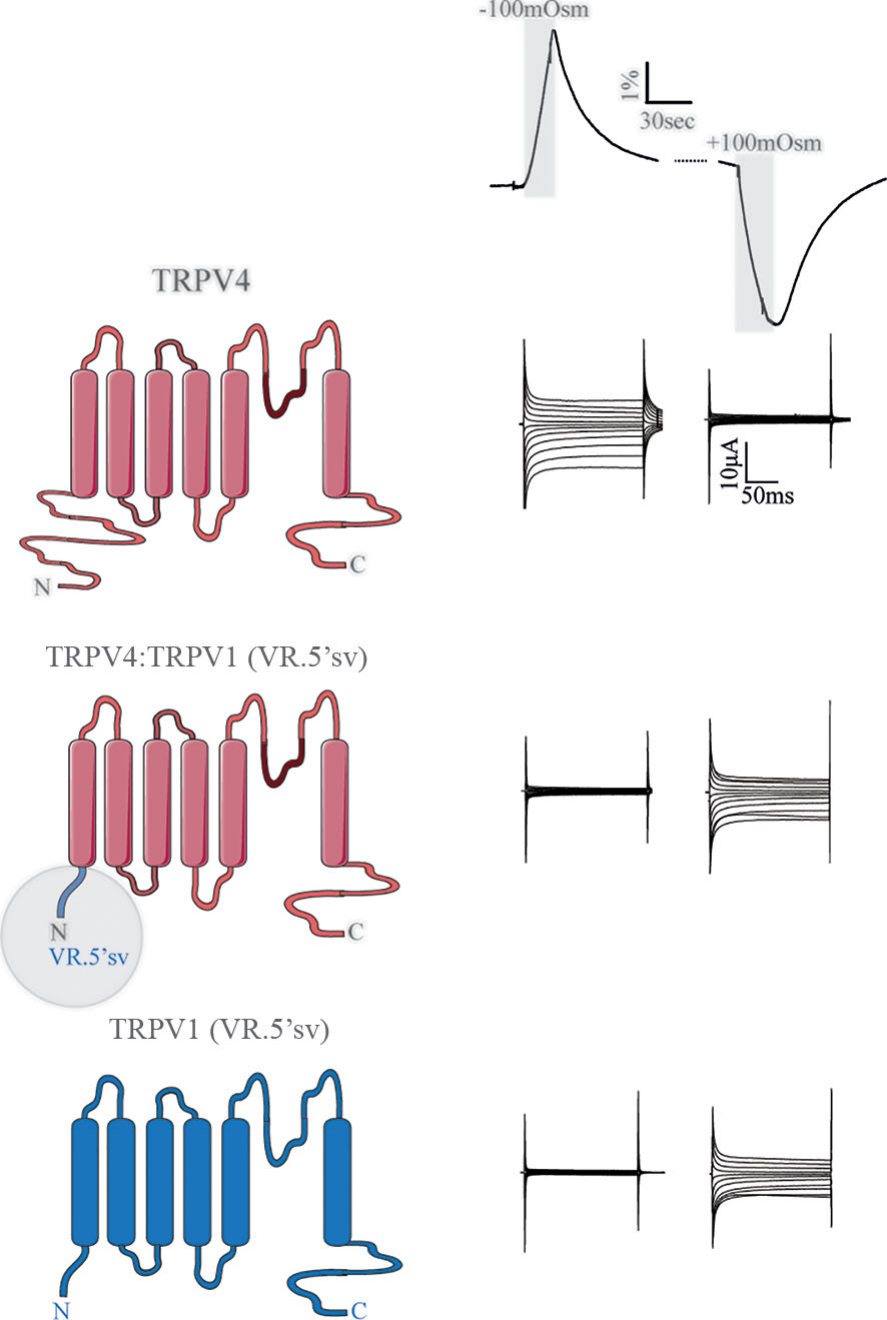
The N-terminus of TRPV4 dictates the directionality of the volume sensing. Channel structures of TRPV4 (top), TRPV4:TRPV1 (VR.5'sv) chimera (with the TRPV4 N terminus replaced by that of VR.5’sv, middle) and VR.5’sv (bottom). The constructs were co-expressed with AQP4 in *Xenopus laevis* oocytes, which were exposed to a hyposmotic or hyperosmotic gradient (Δ100 mOsm) leading to robust cell swelling or cell shrinkage (volume trace, top). TRPV4 responded with augmented membrane currents to a cell volume increase unless its N-terminus was replaced by that of the shrinkage-sensitive VR.5’sv variant of TRPV1 (middle and right panels). Modified from (33).

TRPV4 contains an extensive cytoplasmic N-terminus that contains ankyrin repeats (59, 60). These protein domains can be potential binding hubs for cytoskeletal components (55, 56) and various proteins and small ligands (61). In addition to the ankyrin repeats, the proline-rich region of the N-terminus interacts with the SH3 domain of PACSINs, proteins involved in vesicular membrane trafficking and endocytosis (62, 63). The TRPV4 N-terminus could thus serve as an essential structural element coupling cell volume changes to TRPV4 channel gating. Full deletion of the TRPV4 N-terminus rendered the channel non-functional (33). However, replacing the N-terminus with that of the shrinkage-sensitive variant of the related TRPV1 (the splice variant VR.5’sv) (64) converted the chimeric TRPV4 channel into a sensor of cell shrinkage rather than a sensor of cell swelling, **Figure 4** (33). The N-terminus of these TRP channels thus dictates the volume-sensitivity of the individual channels, with the distal proline-rich domain serving as a key structural element in the process (33).

#### Phosphorylation of TRPV4 Is Not Required for Volume-Sensitivity

**Figure 5:**
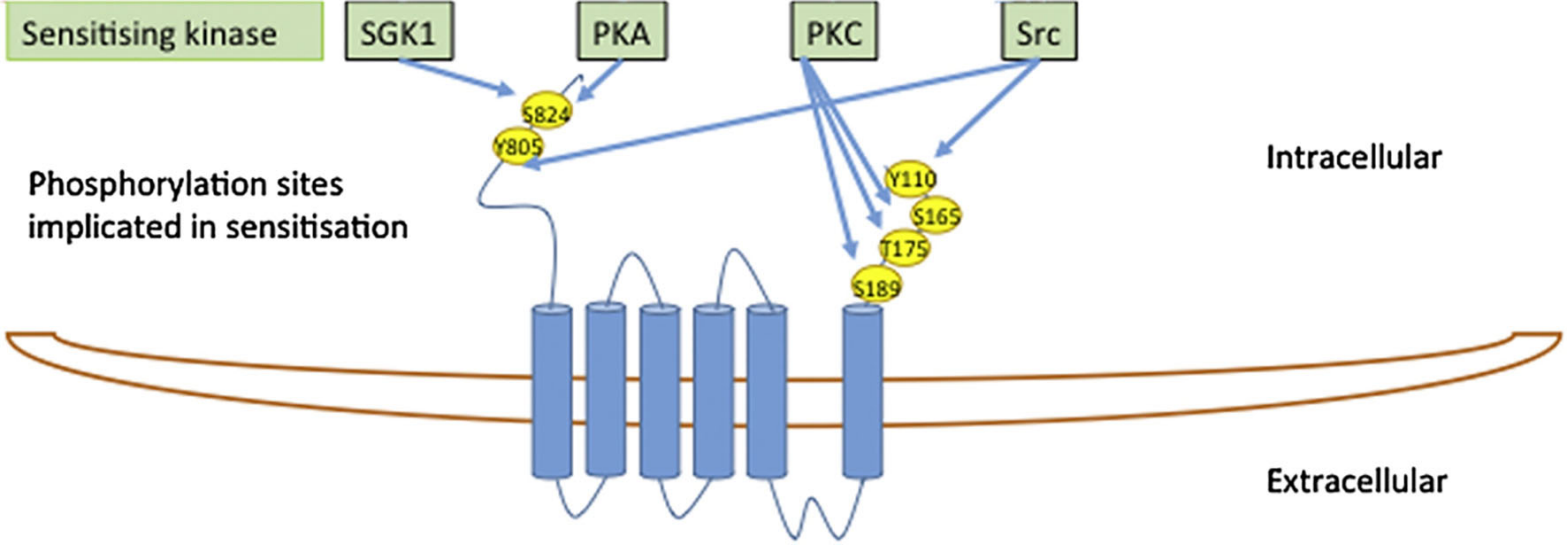
The TRPV4 N- and C-termini contain an abundance of consensus sites for protein kinases. Modified from (65) with permission.

The TRPV4 N and C termini contain an abundance of consensus sites for protein kinases, **Figure 5** (65, 66) and, in addition, serve as anchors for regulatory kinase complexes (54). Some of these kinases may modulate basal TRPV4 activity, rather than directly activate the channel, by altering channel sensitization (66). Such increased channel sensitivity was observed with cell swelling-induced activation of TRPV4 following PKC and Src kinase activity (66, 67). Nevertheless, cell volume-dependent activation of TPV4 occurred readily in the absence of protein kinase activity (PKA, PKC, or PKG), and this cell swelling-induced channel activation regime therefore does not require phosphorylation events (33).

### Indirect Coupling of Cell Volume Changes to TRPV4 Activation

#### Phospholipase A2 and Epoxyeicosatrienoic Acid Metabolites

The molecular coupling from cell swelling to TRPV4 activation may require intermediate steps involving swelling-mediated enzyme activation. Phospholipase A2 (PLA_2_) is activated by large cell volume increases occurring following experimental exposure of the cells to substantial osmotic challenges of up to 200 mOsm (68–71). Swelling-induced PLA_2_ activation promotes occurrence of anandamide and its metabolite arachidonic acid. Subsequent cytochrome P450 epoxygenase-dependent formation of epoxyeicosatrienoic acids may lead to TRPV4 channel opening (72–74), possibly *via* their direct interaction with a binding pocket on TRPV4 (75). Such PLA_2_ activity appeared essential for cell swelling-induced TRPV4 activation in Müller glia and TRPV4-expressing HEK293 cells (18, 33, 34, 72–74). However, in other cell types, i.e. retinal ganglion neurons, sensory neurons, TRPV4-expressing *Xenopus laevis* oocytes or yeast, cell swelling-mediated TRPV4 activation occurred readily in the absence of PLA_2_ activity (30, 31, 33, 41, 76), suggesting that TRPV4 can be directly activated by cell swelling irrespective of PLA_2_ enzymatic products. Curiously, experimental application of downstream products of PLA_2_ enzyme activation, such as 5’,6’-epoxyeicosatrienoic acids, directly activate TRPV4 (in the absence of cell swelling) both in its native setting of Müller glia and upon heterologous expression in HEK293 cells (18, 34). In other cell types, i.e. retinal ganglion neurons and TRPV4-expressing oocytes, these downstream metabolites of the PLA_2_ signaling pathway (e.g. oleic acid, anandamide, 5’,6’-epoxyeicosatrienoic acids) fail to activate TRPV4 (31, 33, 34). PLA_2_ activity thus modulates TRPV4 channel opening differentially in distinct cell types and appears to be a requirement for cell swelling-induced activation of TRPV4 in cell types that permit direct activation of TRPV4 by the PLA_2_ products and metabolites thereof.

## TRPV4 Modulation by Inflammatory Mediators and Other Stimuli

**Figure 6:**
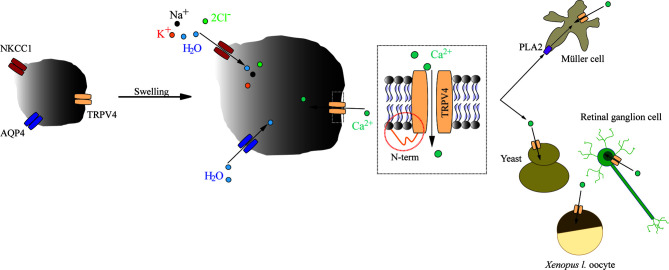
A schematic depicting the mechanisms underlying swelling-induced TRPV4 activation. Increased cell volume activates TRPV4 irrespective of the molecular mechanism underlying the cell swelling. The TRPV4-mediated response to cell volume changes is dictated by its distal-most part of the N terminus, with cell-specific requirement for PLA_2_ activity as permissive for swelling-induced activation of TRPV4.

TRPV4 has been proposed a key role in the response mechanism to pathological events, with excessive TRPV4-mediated Ca^2+^ influx possibly driving reactive gliosis and glial cytokine release (34, 77), and predisposing cells to activation of Ca^2+^-dependent pro-apoptotic signaling cascades (34). Inflammatory mediators are released during activation of inflammatory signaling pathways. A selection of such proinflammatory mediators (TNF-α, IL-1β, TGF-β1) was demonstrated to diminished TRPV4 function after prolonged (24h), but not acute, exposure (78). Inflammatory markers thus join the growing list of TRPV4 modulators, which includes plant extracts such as bisandrographolide and citric acid, apigenin (4’5,7-trihydroxyflavone), a flavone found in many plants (79), RN-1747 (80), dimethylallyl pyrophosphate, an intermediate in the cholesterol synthesis pathway (81), phorbol esters (17, 74, 79), but see (82), and the synthetic lipid GSK1016790A (32, 33). GSK1016790A promotes an open channel conformation similar to that obtained following cell volume-dependent TPV4 activation, suggesting that GSK1016790A stimulates TRPV4 opening in a manner similar to that of swelling-induced channel activation (32). In addition to the cell swelling-mediated activation of TRPV4 and the above-mentioned molecular TRPV4 mediators, TRPV4 senses temperature changes, mechanical stimuli, and flow-related sheer-stress [for review, see (29)], underscoring the polymodality of TRPV4 activation (**Figure 6**).

## Conclusion

In summary, TRPV4 is a genuine sensor of volume changes rather than an osmo-sensor, and is activated by increased cell volume irrespective of the molecular mechanism underlying swelling. The molecular mechanisms that couple altered cell volume to gating of TRPV4 remain obscure, although its distal N-terminus appears to be involved in dictating the volume response (**Figure 6**). Some of the experimental discrepancies over the years regarding TRPV4 activation may originate in cell-specific requirements of volume-dependent activation of TRPV4. Future experimental efforts may reveal how this cell type-specific response is orchestrated.

## Perspective

The polymodality of the TRPV4 remains a topic of continued fascination in the scientific field. The lists of TRPV4-activating stimuli and protein-protein interaction partners rapidly grow. We believe that the field needs to identify which of these are physiologically relevant (perhaps even additive) and which are curious biophysical phenomena, which may never occur in physiology or pathophysiology. The latter may arise due to technical issues in the experimental design i.e. large osmotic gradients, excessively high concentrations of stimulants (which may even be synthetic), extensive mechanical insults, etc. If TRPV4, in the end, is cemented as a true volume sensor in physiological settings, it will be highly relevant to determine the molecular link between volume changes and channel activation. It follows that a revelation of the cellular implications of swelling-activated TRPV4 activation must be resolved; does TRPV4 activation aid the return to the original cell volume or does it in fact worsen the outcome of the cell swelling by promoting a Ca^2+^ overload? We anticipate future exploration of these outstanding research questions alongside the clear definition of TRPV4’s role in diverse human diseases.

## Author Contributions

TLTB drafted the manuscript. TLTB and NM edited and revised the manuscript. All authors contributed to the article and approved the submitted version.

## Funding

The research conducted by the authors was funded (to TLTB) by the Lundbeck Foundation (R208-2015-2859).

## Conflict of Interest

The authors declare that the research was conducted in the absence of any commercial or financial relationships that could be construed as a potential conflict of interest.

## Publisher’s Note

All claims expressed in this article are solely those of the authors and do not necessarily represent those of their affiliated organizations, or those of the publisher, the editors and the reviewers. Any product that may be evaluated in this article, or claim that may be made by its manufacturer, is not guaranteed or endorsed by the publisher.
